# Involvement of Host ATR-CHK1 Pathway in Hepatitis B Virus Covalently Closed Circular DNA Formation

**DOI:** 10.1128/mBio.03423-19

**Published:** 2020-02-18

**Authors:** Jun Luo, Laurie Luckenbaugh, Hui Hu, Zhipeng Yan, Lu Gao, Jianming Hu

**Affiliations:** a Department of Microbiology and Immunology, The Pennsylvania State University College of Medicine, Hershey, Pennsylvania, USA; b Roche Innovation Center Shanghai, Shanghai, China; Virginia Polytechnic Institute and State University

**Keywords:** ATR, CCC DNA, CHK1, DNA damage checkpoint, DNA damage repair, cccDNA, covalently closed circular DNA, hepadnavirus, hepatitis B virus

## Abstract

The covalently closed circular (CCC) DNA of hepatitis B virus (HBV) functions as the only viral transcriptional template capable of producing all viral RNA species and is essential to initiate and sustain viral replication. CCC DNA is converted from a relaxed circular (RC) DNA, in which neither of the two DNA strands is covalently closed. As RC DNA mimics damaged cellular DNA, the host cell DNA damage repair (DDR) system is thought to be responsible for HBV CCC DNA formation. The potential role of two major cellular DDR pathways, the ataxia telangiectasia mutated (ATM) pathway and the ATM and Rad3-related (ATR) pathway, in HBV CCC DNA formation was thus investigated. Inhibition, or expression knockdown, of ATR and its downstream signaling factor CHK1, but not of ATM, decreased CCC DNA formation during *de novo* HBV infection, as well as intracellular CCC DNA amplification, when RC DNA from extracellular virions and intracellular nucleocapsids, respectively, is converted to CCC DNA. Furthermore, a novel RC DNA processing product with 5′ truncated minus strands was detected when the ATR-CHK1 pathway was inhibited, further indicating that this pathway controls RC DNA processing during its conversion to CCC DNA. These results provide new insights into how host cells recognize and process HBV RC DNA in order to produce CCC DNA and have implications for potential means to block CCC DNA production.

## Abstract (precis)

Hepatitis B virus (HBV) chronically infects hundreds of millions of people and remains a major cause of viral hepatitis, cirrhosis, and liver cancer. HBV persistence is sustained by a viral nuclear episome that directs all viral gene expression needed to support viral replication. The episome is converted from an incomplete DNA precursor in viral particles in an ill-understood process. We report here that the incomplete DNA precursor is recognized by the host cell in a way similar to the sensing of damaged cellular DNA for subsequent repair to form the nuclear episome. Intense efforts are ongoing to develop novel antiviral strategies to eliminate CCC DNA so as to cure chronic HBV infection. Our results here provide novel insights into, and suggest novel ways of perturbing, the process of episome formation. Furthermore, our results inform mechanisms of cellular DNA damage recognition and repair, processes essential for normal cell growth.

## INTRODUCTION

**FIG 1:**
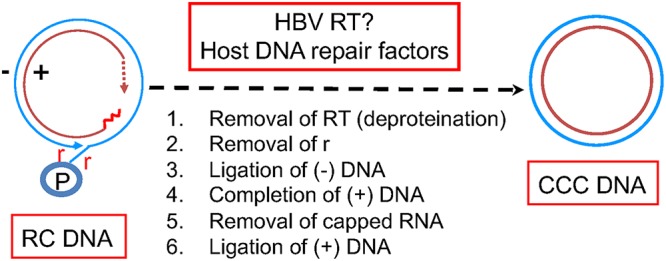
HBV CCC DNA formation from RC DNA. The structures of the HBV RC and CCC DNA are depicted schematically. For the RC DNA, the covalently attached RT protein (P) to the 5′ end of the minus strand (outer circle), the terminal repeats (r) at both ends of the minus strand, the RNA oligomer (the short wavy line) attached to the 5′ end of the plus strand (inner circle), and the heterogeneous and incomplete 3′ end (the dashed arrow) of the plus strand are highlighted. See the text for details.

Hepatitis B virus (HBV) is the prototype member of the *Hepadnaviridae* family (1). HBV causes acute and chronic hepatitis B; worldwide, more than 250 million chronic HBV carriers are living with dramatically increased risk of liver fibrosis, cirrhosis and hepatocellular carcinoma (HCC) (2). HBV has a small (3.2-kb) DNA genome, the so-called relaxed circular (RC) DNA, in which neither strand is covalently closed (Fig. 1) (3, 4). Once the RC DNA is delivered into the host cell nucleus during infection, it is repaired and converted to a covalently closed circular (CCC) DNA (Fig. 1) (5–7). In addition, progeny RC DNA synthesized in newly formed mature nucleocapsids (NCs) can be delivered to the nucleus to form more CCC DNA via the so-called intracellular amplification or recycling pathway (8–11). CCC DNA is key to HBV persistence by serving as the sole transcription template able to produce all viral RNAs essential for viral replication. Currently approved nucleoside analogue drugs block the DNA synthesis activity of the viral reverse transcriptase (RT) and thus the production of RC DNA from an RNA precursor, the pregenome RNA (pgRNA) (12). However, they have no direct effect on CCC DNA, the persistence of which is the major reason that chronic HBV infection is not yet curable in the vast majority of cases (13, 14).

To date, the mechanism of CCC DNA formation remains to be elucidated. Based on the RC DNA structure (Fig. 1), there are five distinct reactions that may be involved in its conversion to CCC DNA. First is the removal of the covalently attached viral RT protein from the 5′ end of the minus strand of the RC DNA, which is used as a protein primer to initiate minus strand synthesis and remains attached until CCC DNA formation (15). Second is the removal of precisely one copy of the terminal redundancy (r) that are present on both ends of the minus strand, as a result of HBV reverse transcription. Third, plus strand elongation is needed to complete the heterogeneous and incomplete plus strand, the synthesis of which is terminated before completion when the virion is secreted. Fourth is the removal of an RNA oligomer that is used to prime plus strand synthesis and remains attached to the 5′ end of the plus strand. Fifth is the ligation of both DNA strands. The order of these events during CCC DNA formation is not yet clear.

Although it remains possible that the viral RT protein plays a role in CCC DNA formation, e.g., by completing the plus strand in RC DNA, current evidence suggests that the DNA polymerase activity of RT is not essential for this or any other step in CCC DNA formation (16, 17). It remains possible that another yet-be-uncovered activity of RT may be involved. On the other hand, it is generally assumed that the host cell DNA repair machinery is required for CCC DNA formation in HBV-infected cells (6, 7, 18). A DNA polymerase, DNA Pol kappa, was identified as a host factor required for HBV CCC DNA formation, presumably by completing the plus strand in RC DNA (19). A few other host DNA polymerases were also found to play a role in CCC DNA formation (19, 20). Both DNA ligase I and ligase III were reported to play a role in CCC DNA formation, presumably by carrying out the ligation of one or both DNA strands (21). The tyrosyl DNA phosphodiesterase 2 (TDP2), which was identified by its ability to remove topoisomerase II (Topo II) from covalent DNA adducts (22), is able to cleave the viral RT protein off the RC DNA precisely at the phosphodiester bond between the protein and the 5′ end of the minus strand DNA as found between Topo II and covalently attached DNA adducts, but TDP2 is clearly not essential for, and may even suppress, HBV CCC DNA formation (23–26). The Flap endonuclease 1 (Fen-1), known to cleave unannealed 5′ DNA fragment at DNA three strand junctions, was recently reported to play a role in CCC DNA formation (27), presumably by cleaving the 5′ r fragment (perhaps together with the RT protein; Fig. 1) from the 5′ end of the minus strand. Topo I and II have also very recently been reported to play a role in HBV CCC DNA formation (28).

The biochemical pathways of CCC DNA formation also remain to be defined. It is possible that multiple steps listed in Fig. 1 may be carried out simultaneously. For example, it is conceivable that the removal of the RT protein and the 5′ copy of r from the minus strand may be accomplished in one step via an endonucleolytic attack just 3′ of the 5′ r sequence (e.g., by FEN-1, as suggested above). Consistent with the removal of the RT protein being one of the initial steps in CCC DNA formation, a protein-free (PF) or deproteinated (dp) RC (PF-RC or dp-RC) DNA has been identified in cultured cells that support HBV replication (29–31). However, the fine structure of PF-RC DNA, especially at the 5′ end of the minus strand, remains to be characterized and the apparent single band resolved by agarose gel electrophoresis may actually represent multiple related DNA species. Some of those may in fact be dead-end processing products from the (RT-linked) RC DNA (26, 29), whereas others are true intermediates on their way to form CCC DNA. Indeed, we have recently identified a novel form of RC DNA in which the minus strand is covalently closed but the plus strand remains open, the so-called closed minus strand RC (cM-RC) DNA (32). This appears to be the best candidate to date for an authentic intermediate in CCC DNA formation, which would further suggest that minus strand closing occurs before the plus strand during CCC DNA formation. Interestingly, Topo I was reported to be more important than Topo II for this early step of cM-RC DNA formation (28).

Before RC DNA is converted to CCC DNA, it needs to be recognized by the host cell DNA damage sensing mechanisms that, in turn, signal to the downstream effectors (i.e., the actual repair factors such as the DNA polymerases and ligases) to carry out the conversion reaction. In this respect, virtually nothing is currently known. There are three major cellular DNA damage repair (DDR) pathways, the ataxia telangiectasia mutated (ATM) pathway, the ATM and Rad3-related (ATR) pathway (33), and nonhomologous end joining (NHEJ). NHEJ is error-prone (21) and thus, in principle, is unlikely to be involved HBV CCC DNA formation from RC DNA since this process demands high precision to maintain viral replication. Indeed, it has been reported that NHEJ is not needed for HBV RC DNA to CCC DNA conversion, but it is instead involved in the formation of defective CCC DNA carrying imprecise junctions from a minor form of HBV genomic DNA, the double-stranded linear DNA, via circularization (intramolecular ligation) (21, 34, 35).

It is currently unknown whether either the ATR, the ATM, or both pathways are involved in repairing the HBV RC DNA to form CCC DNA. Both ATR and ATM are protein kinases that signal by phosphorylating downstream proteins, such as CHK1 and CHK2, respectively, to ultimately recruit appropriate effectors to repair various DNA damages. In this study, we have begun to examine the role of these host DNA repair pathways using HBV infection and replication systems that allow CCC DNA formation during either *de novo* infection or intracellular CCC DNA amplification. Our results obtained in these systems through the use of multiple specific inhibitors of these pathways and gene expression knockdown support an important role of the ATR, but not the ATM, pathway in HBV CCC DNA formation.

## RESULTS

### Multiple inhibitors of the ATR-CHK1 pathway reduced HBV CCC DNA formation during viral infection in human hepatoma cells.

**FIG 2:**
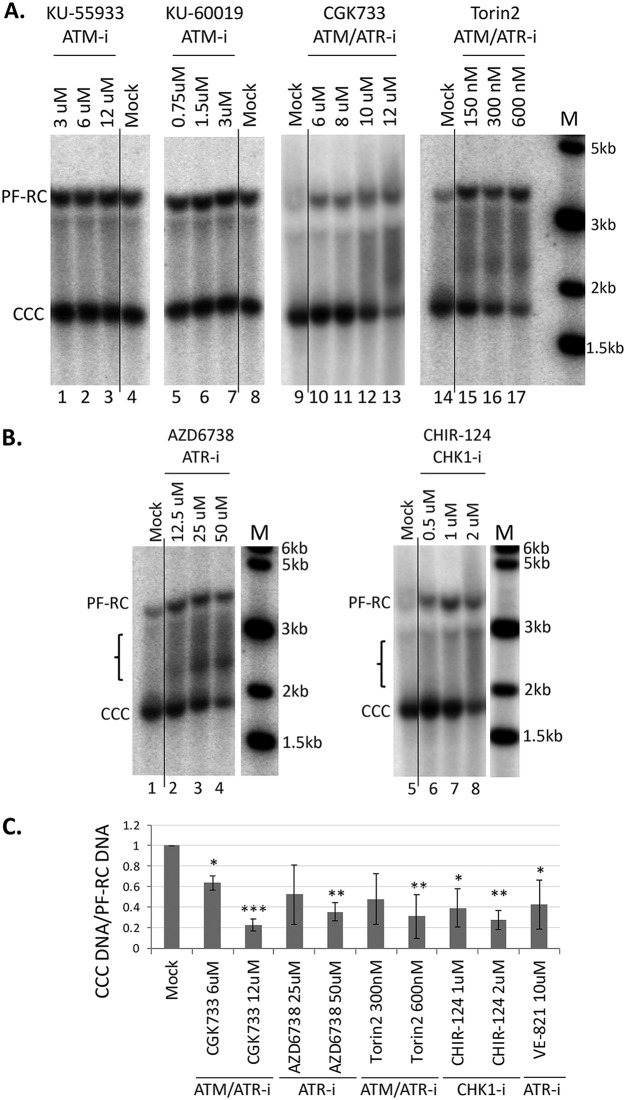
Effects of ATM, ATR, and CHK1 inhibitors on CCC DNA formation during HBV infection in HepG2-NTCP cells. The HepG2-NTCP cells were infected with the HBV inoculum harvested from HepAD38 cells. The following inhibitors were added at the indicated concentrations at the same time as the inoculum and maintained until the cells were harvested: the ATM inhibitors (ATM-i) KU-55933 and KU-60019; the ATM/ATR dual inhibitors (ATM/ATR-i) CGK733 and Torin2; the ATR (ATR-i) inhibitors AZD6738 and VE-821; and the CHK1 inhibitor (CHK1-i) CHIR-124. HBV PF DNA was extracted from the infected cells at 3 days postinfection and measured by Southern blotting using a ^32^P-labeled HBV DNA probe. (A and B) Representative Southern blot autoradiograms. The brackets indicate the putative RC DNA processing products accumulating under conditions of ATR-CHK1 inhibition (see Fig. 8, below). (C). Quantitative analysis of Southern blot results from multiple independent experiments. The data are expressed as CCC DNA levels normalized to those of PF-RC DNA, with the normalized CCC DNA level from the mock-treated cells set to 1.0. *, *P* ≤ 0.05; **, *P* ≤ 0.01; ***, *P* ≤ 0.001. The vertical thin lines in the images denote where the different parts of the same gel, with the same exposure, were spliced together in order to remove other parts of the gel that are not presented here.

Since the HBV RC DNA mimics damaged cellular DNA, the host cell DDR system is thought to be responsible for mediating the repair of HBV RC DNA to form CCC DNA. To test the role of the ATM or ATR pathway in HBV CCC DNA formation, we tested the effects of specific inhibitors of these pathways on HBV CCC DNA formation in human hepatoma HepG2 cells reconstituted with the HBV receptor NTCP, in which HBV CCC DNA can be formed early during *de novo* infection from the incoming virion RC DNA (32). We started with four compounds, the ATM inhibitors KU-55933 and KU-60019, and ATM/ATR dual inhibitors CGK733 and Torin2. HepG2-NTCP cells were infected with HBV, and the inhibitors were added along with the virus. HBV CCC DNA formed from the incoming virion RC DNA was extracted 3 days postinfection and detected by Southern blotting. The results showed that both of the ATM/ATR dual inhibitors decreased the CCC DNA level in a dose-dependent manner, whereas neither of the two ATM inhibitors showed any effect (Fig. 2A and C). This suggested that the ATR, but not the ATM, pathway might be involved in HBV CCC DNA formation during infection. We then tested several additional, structurally distinct inhibitors that target the ATR-CHK1 pathway. The results showed that CCC DNA formation was suppressed by two ATR inhibitors (AZD6738 and VE-821) (Fig. 2B and C) and the CHK1 inhibitor (CHIR-124) (Fig. 2B and C), suggesting that the ATR-CHK1 pathway indeed played a critical role in HBV CCC DNA formation during *de novo* infection. Interestingly, treatment with the ATR and CHK1 inhibitors led to the accumulation of a prominent smear (denoted by a bracket in Fig. 2) representing processing products from RC DNA (see below).

### Inhibition of the ATR-CHK1 pathway suppressed HBV CCC DNA formation during intracellular CCC DNA amplification.

**FIG 3:**
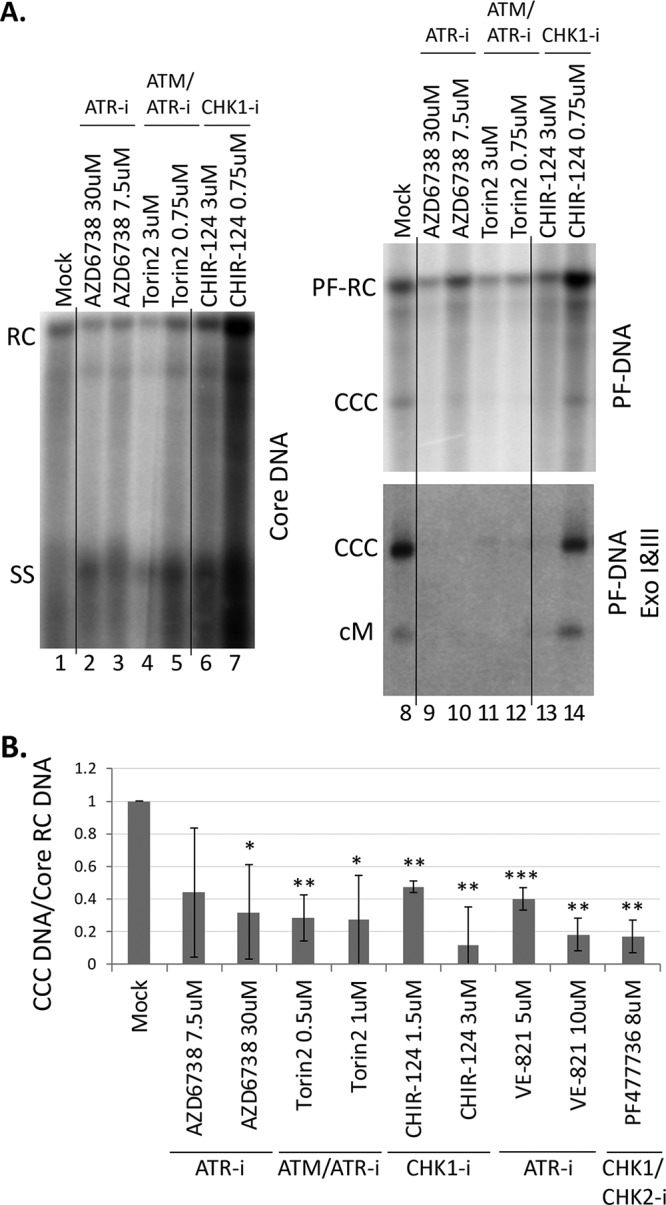
Effects of ATR and CHK1 inhibitors on CCC DNA formation via intracellular amplification in AML12HBV10 cells. HBV replication and CCC DNA formation were induced in AML12HBV10 cells, and the indicated inhibitors were added 4 days after the start of induction. The inhibitors used included the ATM/ATR dual inhibitor Torin2, the ATR inhibitors AZD6738 and VE-821, and the CHK1 inhibitors CHIR-124 and PF477736 at the indicated concentrations. After 3 days of inhibitor treatment (i.e., 7 days of induction), HBV core DNA and PF DNA were extracted from the cells and measured by Southern blotting using a ^32^P-labeled HBV DNA probe. (A) Representative Southern blot autoradiograms of HBV core DNA (lanes 1 to 7) and PF DNA (lanes 8 to 14, top panel). HBV PF DNA was further treated with Exo I and III to remove RC DNA for specific detection of CCC DNA (lanes 8 to 14, bottom panel). cM, covalently closed minus strand DNA. (B) Quantitative analysis of Southern blot results from multiple independent experiments. The data are expressed as CCC DNA levels normalized to those of core RC DNA, with the normalized CCC DNA level from the mock-treated cells set to 1.0. *, *P* ≤ 0.05; **, *P* ≤ 0.01; ***, *P* ≤ 0.001. The vertical thin lines in the images denote where the different parts of the same gel, and with the same exposure, were spliced together in order to remove other parts of the gel that are not presented here.

We tested the effects of the ATR-CHK1 inhibitors in a system different from the above-described HepG2-NTCP HBV infection system. We have recently reported that a mouse hepatocyte-derived cell line (AML12HBV10), in contrast to normal mouse hepatocytes (36) or other mouse hepatocyte cell lines tested so far (37, 38), has the ability to support HBV CCC DNA formation via the intracellular amplification pathway from RC DNA in mature NCs formed intracellularly (39). As shown in Fig. 3, specific inhibitors of ATR/ATM (Torin2), ATR (AZD6738 and VE-821), or CHK1 (CHIR-124 and PF477736) decreased CCC DNA formation in these cells, suggesting that the ATR-CHK1 pathway also played a critical role in HBV CCC DNA formation via the intracellular CCC DNA amplification pathway. The levels of PF-RC DNA, which is also derived from RC DNA in mature NCs, generally paralleled those of RC DNA in NCs (Fig. 3), indicating no significant effect of the inhibitors on PF-RC DNA formation. For reasons not yet clear, some inhibitors apparently affected the levels of core DNA. In particular, the CHK1 inhibitor CHIR-124 significantly increased the core DNA levels at the lower concentration (0.75 μM) but not at the higher concentration (3 μM) (Fig. 3A, lanes 6 and 7). We have recently detected a likely intermediate in the conversion of HBV RC DNA to CCC DNA, cM-RC DNA, which can be revealed by Exo I and Exo III digestion that degrades the open plus strand and thus generates closed minus strand DNA (32). We found that the levels of cM-RC DNA were reduced in parallel to those of CCC DNA when the ATR-CHK1 pathway was inhibited (Fig. 3A), which suggested that the ATR-CHK1 pathway was important for this early step in CCC DNA formation.

**FIG 4:**
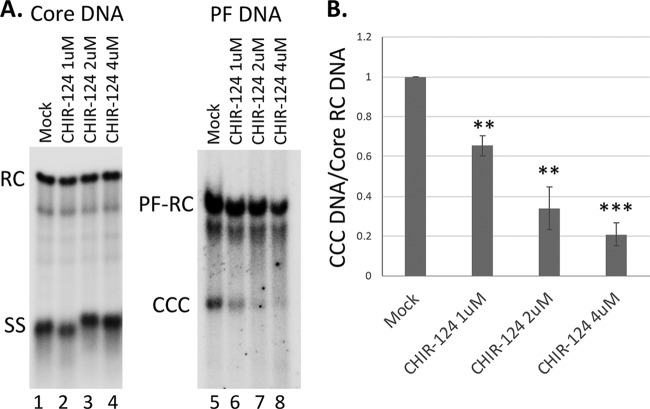
Effect of CHK1 inhibitor on CCC DNA formation via intracellular amplification in HepAD38 cells. HBV pgRNA transcription were induced in HepAD38 cells by Tet removal. To accumulate SS HBV DNA, PFA was added into the culture medium on day 2 after Tet removal and maintained for the next 4 days. PFA was then removed to allow the synthesis of RC DNA and the formation of CCC DNA. At the time of PFA removal, the CHK1 inhibitor CHIR-124 was added. Twenty-four hours later, HBV core DNA and PF DNA were extracted from the cells and measured by Southern blotting using a ^32^P-labeled HBV DNA probe. (A) Representative Southern blot autoradiograms of HBV core DNA (lanes 1 to 4) and PF DNA (lanes 5 to 8). (B) Quantitative analysis of Southern blot results from multiple independent experiments. The data are expressed as CCC DNA levels normalized to those of core RC DNA, with the normalized CCC DNA level from the mock-treated cells set to 1.0. **, *P* ≤ 0.01; ***, *P* ≤ 0.001.

We also adopted the synchronized CCC DNA formation system using induced HepAD38 cells, as recently reported (28), which allows rapid HBV CCC DNA formation via intracellular amplification within 1 day (see Materials and Methods for details). This rapid CCC DNA synthesis system helps to avoid potential cytotoxic or pleiotropic effects associated with prolonged DDR inhibition. As shown in Fig. 4, the CHK1 inhibitor CHIR-124 was able to suppress HBV CCC DNA formation in a dose-dependent manner, in this system as in the AML12HBV10 system above.

### Inhibition of the ATR-CHK1 pathway suppressed HBV CCC DNA formation in PHHs and PXB cells during HBV infection.

**FIG 5:**
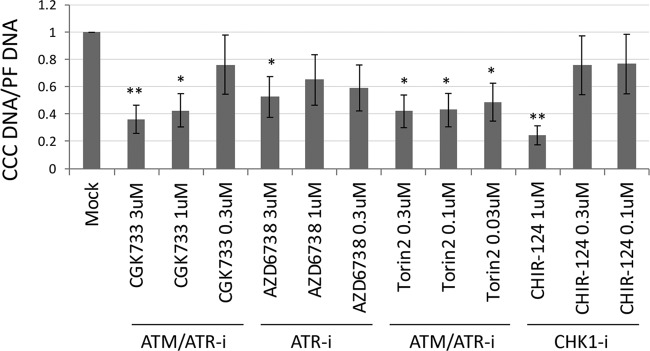
Effects of ATR and CHK1 inhibitors on CCC DNA formation during HBV infection in PHHs. The PHHs were infected with HBV and treated with the indicated inhibitors at the same time. The inhibitors used included the ATM/ATR dual inhibitors CGK733 and Torin2, the ATR inhibitor AZD6738, and the CHK1 inhibitor CHIR-124 at the indicated concentrations. HBV PF DNA was extracted from the cells 3 days after infection. qPCR was used to quantify the CCC DNA using CCC DNA specific primers or total HBV PF DNA using the total DNA primers. The data are expressed as CCC DNA levels normalized to total PF DNA, with the normalized CCC DNA level from the mock-treated cells set to 1.0. *, *P* ≤ 0.05; **, *P* ≤ 0.01.

**FIG 6:**
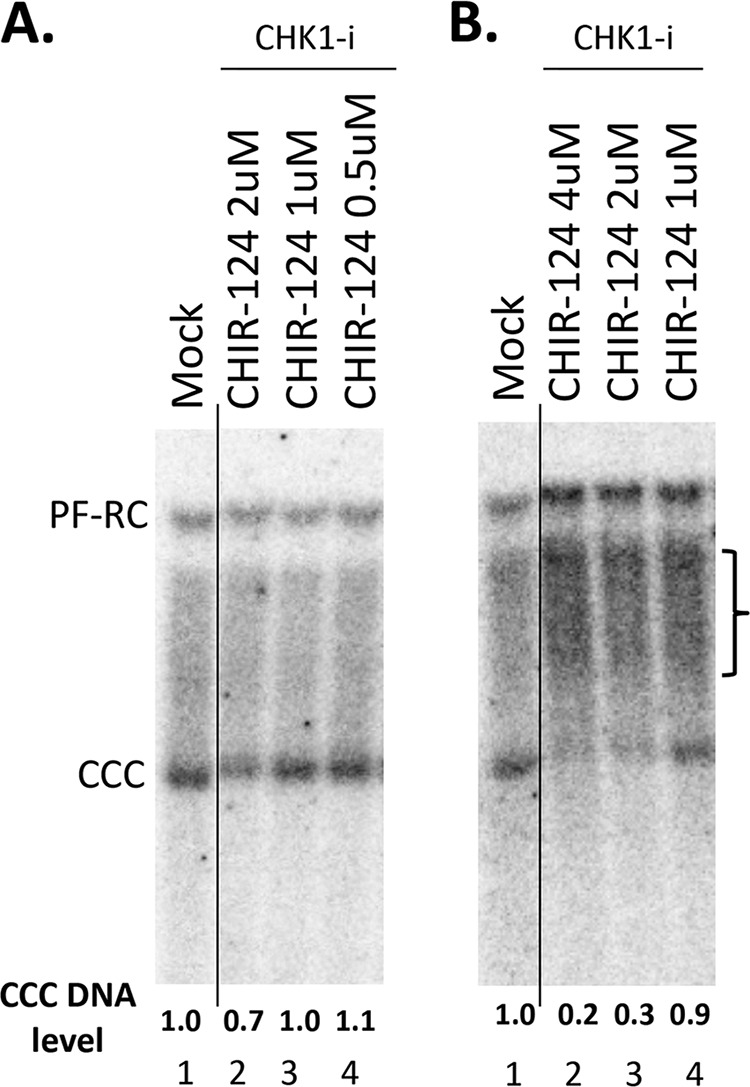
Effects of ATR and CHK1 inhibitors on CCC DNA formation during HBV infection in PXB cells. The PXB cells were infected with HBV and treated with the CHK1 inhibitor CHIR-124 at the indicated concentrations. HBV PF DNA was extracted from the cells 3 days after infection and measured by Southern blotting using a ^32^P-labeled HBV DNA probe. (A and B) Representative Southern blot autoradiograms of PF DNA extracted from two different batches of PXB cells infected with HBV. The brackets indicate the putative RC DNA processing products accumulating under conditions of CHK1 inhibition (see Fig. 7 below). The CCC DNA levels are indicated at the bottom, with that from the mock-treated cells set to 1.0. The vertical thin lines in the images denotes where the different parts of the same gel, and with the same exposure, were spliced together in order to remove other parts of the gel that are not presented here.

We tested the effects of ATR-CHK1 inhibitors on HBV CCC DNA formation during HBV infection of primary human hepatocytes (PHHs), which is thought to mimic best the authentic host cells of HBV infection, i.e., hepatocytes in the human liver (40). Again, the results showed that multiple inhibitors of the ATR-CHK1 pathway could decrease HBV CCC DNA formation in PHHs during HBV infection (Fig. 5), including the ATM/ATR dual inhibitors CGK733 and Torin2, the ATR inhibitor AZD6738, and the CHK1 inhibitor CHIR-124. Similarly, we found that the CHK1 inhibitor (CHIR-124) could decrease CCC DNA formation during HBV infection in two different batches of PXB cells (Fig. 6), which are freshly prepared human hepatocytes harvested from the chimeric mice with humanized livers (41).

### CHK1 siRNA knockdown could decrease CCC DNA formation during HBV infection.

**FIG 7:**
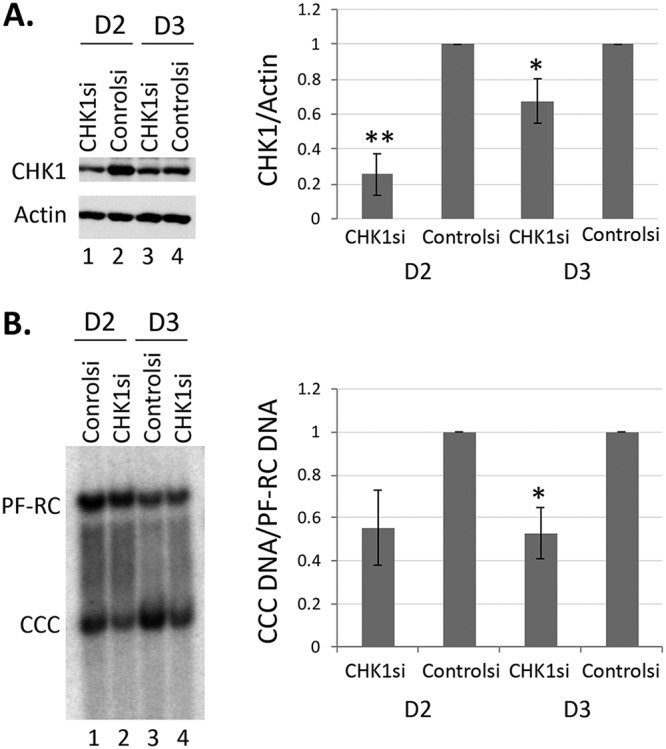
Effect of CHK1 siRNA knock down on CCC DNA formation during HBV infection in HepG2-NTCP cells. The HepG2-NTCP cells were transfected with the CHK1 siRNA (CHK1si) or negative-control siRNA (Controlsi). Subsequently, they were infected with HBV. Cells were harvested for analysis of CHK1 protein levels by Western blotting (A) and HBV PF DNA by Southern blotting (B) on day 2 and day 3 postinfection. β-Actin was used as a loading control for Western blot analysis in panel A. HBV PF DNA was detected with a ^32^P-labeled HBV DNA probe in panel B. Quantitative results from multiple independent experiments were shown to the right. The data are expressed as CHK1 protein levels normalized to actin (A) or CCC DNA levels normalized to PF-RC DNA (B), with the normalized CHK1 protein or CCC DNA level from the mock-treated cells set to 1.0. *, *P* ≤ 0.05; **, *P* ≤ 0.01.

To confirm the results obtained above using small molecule inhibitors, we performed small interfering RNA (siRNA) knockdown to suppress the ATR-CHK1 pathway during HBV infection in HepG2-NTCP cells. Knockdown of CHK1 expression (by 40 to 80%) was confirmed by Western blotting (Fig. 7A). Southern blot analysis showed that CCC DNA levels were decreased by ca. 50% in CHK1 knockdown HepG2-NTCP cells (Fig. 7B), confirming the role of CHK1 in supporting HBV CCC DNA formation during HBV infection.

### Inhibition of the ATR-CHK1 pathway induced accumulation of novel processing product from RC DNA.

**FIG 8:**
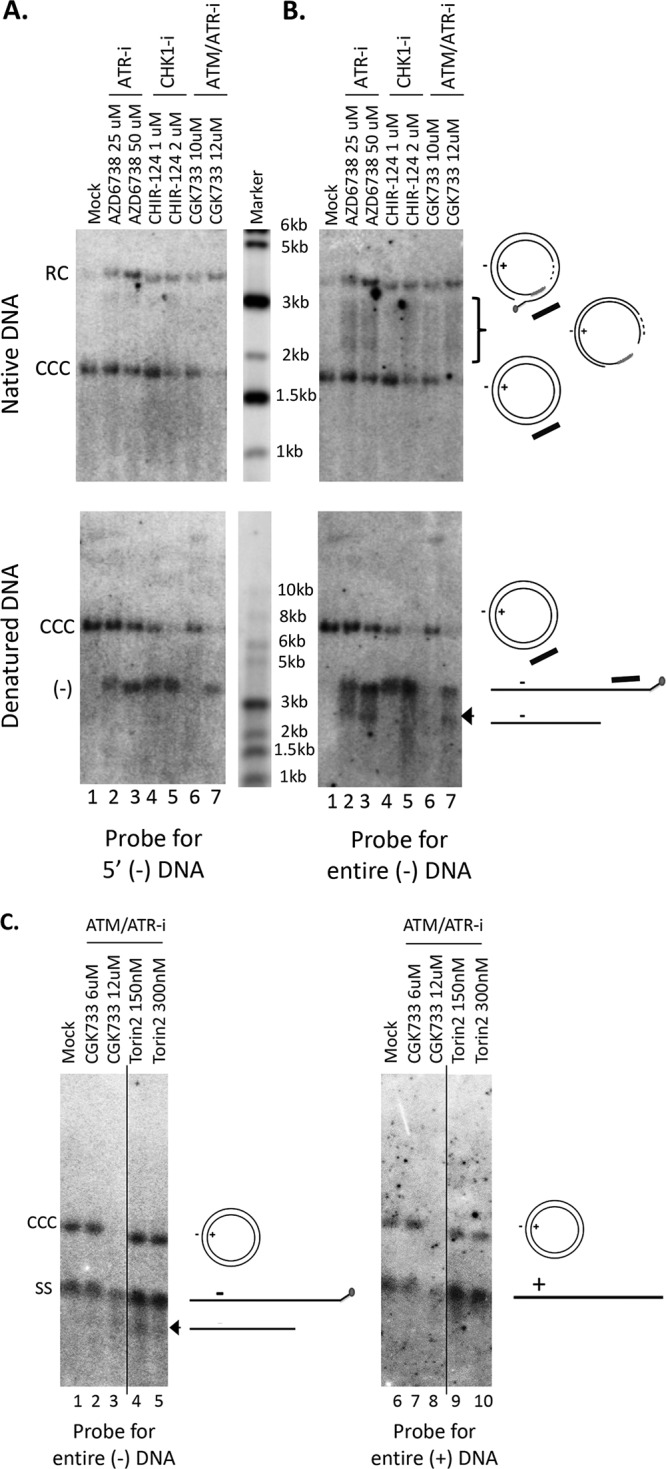
Accumulation of 5′ truncated minus strands under conditions of ATR-CHK1 inhibition. HBV PF DNA extracted from HBV-infected HepG2-NTCP cells, either mock treated or treated with the indicated inhibitors, was analyzed by Southern blotting using a riboprobe hybridizing to the 5′ end of the viral minus strand DNA (A), to the full-length minus strand DNA (B; C, lanes 1 to 5), or to the full-length plus strand DNA (C, lanes 6 to 10). Native DNA (undenatured) was used for the results shown in the top of panels A and B and heat denatured (95°C, 10 min) DNA at the bottom of panels A and B and in panel C. The diagrams to the right of the images depict the various HBV DNA forms detected on the Southern blots, including the RC DNA, CCC DNA, full-length minus strand DNA (–), full-length plus strand DNA (+), RC DNA processing products missing the 5′ end of the minus strand (bracket), and the 5′ truncated minus strand DNA (arrowhead, B, bottom; and C). The bracket indicates the RC DNA processing products accumulating under conditions of ATR-CHK1 inhibition. The short black bar represents the riboprobe for the specific detection of the 5′ end of the minus strand DNA; it specifically hybridizes to the minus strand DNA sequence from nt 1377 to 1805. The vertical thin lines in the images denote where the different parts of the same gel, and with the same exposure, were spliced together in order to remove other parts of the gel that are not presented here.

An increase in PF-RC DNA was observed when the cells were treated with inhibitors of the ATR-CHK1 pathway (Fig. 2A, lanes 10 to 13 and 15 to 17; Fig. 2B, lanes 2 to 4 and 6 to 8), which is consistent with the notion that at least some of the PF-RC DNA functioned as a precursor to CCC DNA whose conversion to CCC DNA was suppressed by the inhibitor treatment. Furthermore, inhibition of the ATR-CHK1 pathway induced a prominent smear running between the RC and CCC DNA in the HBV-infected HepG2-NTCP cells (Fig. 2A, lanes 12, 13, and 15 to 17; Fig. 2B, lanes 2 to 4 and 6 to 8; Fig. 8B, top, lanes 2 to 7), suggesting that novel processing products of RC DNA might have accumulated when the ATR-CHK1 pathway was inhibited and the conversion of RC DNA to CCC DNA was reduced. The increased accumulation of PF-RC DNA and the putative RC-DNA processing intermediates by inhibition of the ATR-CHK1 pathway further helped to exclude the possibility that the decrease in CCC DNA caused by ATR-CHK1 inhibition was due to gross cytotoxicity leading to cell death, which would have resulted in loss of PF-RC DNA as well and no accumulation of any RC DNA processing. To determine the structures of these novel processing products, we used strand-specific riboprobes that targeted different regions of the minus or plus strand of RC DNA for Southern blot analysis of native (Fig. 8A and 8B, top) and heat-denatured PF DNA (Fig. 8A and 8B, bottom). The results showed that these novel processing products lacked the 5′ end of the minus strand (by up to ca. 1,000 nucleotides [nt]). Thus, we could detect the DNA smear between the RC and CCC DNA in the native DNA samples and the minus strand DNA species running below the full-length minus strands in denatured DNA samples using the riboprobe targeted to the entire length of minus strands (Fig. 8B and C) but not that to the 5′ end of minus strands (Fig. 8A). Further experiments showed that these processing products retained the 3′ ends of minus strands and full-length plus strands (Fig. 8C).

## DISCUSSION

Our results indicate that ATR is the major cellular DDR pathway that is likely involved in HBV CCC DNA formation from RC DNA, a critical process for the establishment and maintenance of HBV infection. The involvement of ATR in RC DNA to CCC DNA formation is consistent with the structure of the HBV RC DNA. Although neither of the two strands in RC DNA is covalently closed, the two discontinuities are separated by a region more than 200 bp long, which harbors the complementary sequences located at the 5′ ends of both DNA strands holding the RC DNA in a circular configuration (Fig. 1). Thus, no true double-stranded break (DSB) exists in RC DNA, which is known to trigger the ATM pathway (33). Instead, SS DNA that lies adjacent to DS DNA, as found in RC DNA (i.e., where only the minus strand but not the plus strand sequence is present, Fig. 1), is the main trigger of ATR activation (33, 42) (see further discussion on ATR induction below).

**FIG 9:**
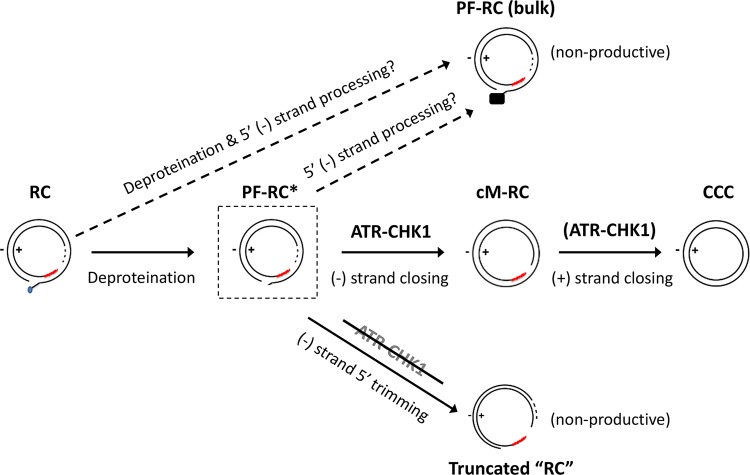
Role of the ATR-CHK1 pathway in RC DNA processing and CCC DNA formation. Removal of the RT protein (deproteination) from the 5′ end of the minus stand DNA may be carried out by multiple competing pathways, with some being nonproductive for CCC DNA formation and independent of the ATR-CHK1 pathway (e.g., the production of the bulk of PF-RC DNA detected in established cell lines) (top). For productive CCC DNA formation (middle), the early stage of cM-RC DNA production requires the ATR-CHK1 pathway. cM-RC DNA is, in turn, presumably derived from a hypothetic (not yet detected) PF-RC DNA intermediate (PF-RC*; in dashed box) in which the RT protein has been removed. PF-RC* DNA may also be converted to the bulk PF-RC DNA in cell lines via further processing at the 5′ end of the minus strand, which helps to stabilize and allows the accumulation of the bulk PF-RC DNA (middle and top). The solid black box on the bulk PF-RC DNA (top) denotes the unknown nature of the 5' end of the minus strand. The ATR-CHK1 pathway is proposed to facilitate the conversion of PF-RC* DNA to cM-RC DNA (by recruiting necessary repair effector enzymes such as a DNA ligase) and, possibly, the subsequent conversion of cM-RC DNA to CCC DNA (by recruiting effectors to process and close the plus strand). When the ATR-CHK1 pathway is inhibited (bottom), extensive minus strand 5′ trimming of PF-RC* DNA is triggered, which would linearize the RC DNA (hence “RC” is in quotation marks) and render it incapable of further conversion to CCC DNA. See the text for details.

In contrast to the significant reduction of CCC DNA formation, no decrease in the bulk PF-RC DNA was observed when the ATR-CHK1 pathway was inhibited, indicating that RC DNA deproteination, or at least deproteination and any other processing events that generate the bulk (stable) of PF-RC DNA, presumably carried out by TDP2 and/or FEN1 (and likely additional factors) as introduced earlier, occur independent of the ATR-CHK1 pathway (Fig. 9, top). This result is also consistent with the notion that the bulk of PF-RC DNA may be a dead-end processing product from RC DNA that accumulates in hepatoma cell cultures (but not *in vivo*) and is incapable of conversion to CCC DNA, as we proposed earlier (26, 32). On the other hand, cM-RC DNA was reduced to a similar degree as CCC DNA when the ATR-CHK1 pathway was inhibited, suggesting a role for the ATR-CHK1 pathway in the minus strand closing process and also supporting the notion that cM-RC DNA is a true precursor to CCC DNA and an authentic intermediate during the conversion of RC DNA to CCC DNA (Fig. 8, middle) (32).

While some trimming of the 5′ end of the minus strand of RC DNA (e.g., removal of the 5′ copy of r; Fig. 1) may be required for CCC DNA formation, the extensive trimming (by hundreds of nucleotides) of the 5′ end of the minus strand of RC DNA, as we observed here under conditions of ATR-CHK1 inhibition, may be triggered only upon ATR-CHK1 inhibition and is unlikely to be part of the normal process in RC DNA to CCC DNA conversion (Fig. 9, bottom), since extensive trimming would create a true DSB (i.e., linearization of RC DNA) and preclude ligation (i.e., covalent closing) of the minus strand of RC DNA, which, as we have shown recently (32), is likely an early step in CCC DNA formation occurring before plus strand closing. Indeed, extensive 5′ end trimming of the minus strand of RC DNA and formation of DSB may be normally blocked by the ATR-CHK1 pathway, which is known to modulate nuclease activity during DNA repair (33, 42). Thus, one function of the ATR-CHK1 pathway may be to protect the integrity of the RC DNA to allow its conversion to CCC DNA.

How HBV infection/replication triggers activation of the ATR-CHK1 pathway remains to be clarified. In transformed cells in culture such as HepG2 cells, the normal DNA repair pathways may already be misregulated in the absence of HBV replication, rendering it difficult to determine whether HBV infection/replication can trigger these pathways using these systems. Indeed, we found that the ATR-CHK1 pathway appeared to be constitutively activated in HepG2 cells. Thus, in the HepG2-NTCP cells, in the absence of HBV infection or replication, we observed constitutive nuclear pCHK1 staining, which could be blocked, as expected, by inhibitors of the ATR-CHK1 pathway (see Fig. S1A in the supplemental material) but did not appear to be further stimulated by HBV infection under our infection conditions (data not shown). Similarly, pCHK1 induced by UV treatment in AML12HBV10 cells could be suppressed by inhibitors of the ATR-CHK1 pathway, as expected (Fig. S1B). Interestingly, in the immortalized AML12HBV10 cells, which are thought to be nontransformed and mimic normal hepatocytes better than hepatoma cells, we could detect transient induction of the ATR-CHK1 pathway by HBV replication, as indicated by the increase of pCHK1 shown by both immunofluorescence (Fig. S2A) and Western blot analysis (Fig. S2B). Furthermore, induction of CHK1 phosphorylation could also be observed during HBV infection of the PXB cells (Fig. S3). Thus, the ATR-CHK1 pathway could be activated by HBV replication at least under some conditions. However, we noticed that the induction of CHK1 phosphorylation during HBV replication was not always detected; we observed this phenomenon in some but not all experiments in AML12HBV10 cells for reasons not yet understood. It is possible that the induction of the ATR-CHK1 pathway by HBV in these cells was very transient under some conditions or that CHK1 activation was dependent on some yet-to-be defined factors. Interestingly, HBV activation of ATR and CHK1 has been reported previously although the precise mechanisms remains unclear (43–45). In particular, the viral X protein (HBx) has been suggested to activate the ATR-CHK1 pathway. Therefore, HBV may be able to activate this cellular pathway via multiple mechanisms, including the putative role of the RC DNA structure (i.e., SS DNA adjacent to DS DNA) and the HBx protein.

10.1128/mBio.03423-19.1 FIG S1 Inhibition of CHK1 phosphorylation in HepG2-NTCP and AML12HBV10 cells by ATR-CHK1 inhibitors. The HepG2 NTCP cells were mock treated or treated with the indicated compounds (the ATM/ATR dual inhibitor CGK337, the ATR inhibitor AZD6738, and CHK1 inhibitor CHIR-124) overnight before immunofluorescence staining using the pCHK1 antibody (S317, CST12302). The DAPI staining shows the cell nucleus. Staining was performed on 35-mm glass-bottom tissue culture dishes (MatTek), and the images were collected using a Leica SP8 confocal microscope. (B) The AML12HBV10 cells were mock treated or treated with the indicated compounds (KU-55933, 6 μM; KU-60019, 6 μM; AZD6738, 30 μM; CHIR-124, 3 μM; and Torin2, 3 μM) for overnight before exposing to UV light (120 J/m^2^) to induce the DNA damage response. Fresh culture medium was then added back to the irradiated cells, and the culture was continued for another three hours before total cell lysate was prepared. SDS-PAGE and Western blot analysis were performed to detect the pCHK1, total CHK1, pCHK2, and total CHK2 proteins using the pCHK1 antibody (S317; CST12302; Cell Signaling Technology), pCHK2 antibody (T68; CST2661; Cell Signaling Technology), total CHK1 antibody (AM7401a; Abgene), and total CHK2 antibody (AP4999a; Abgene). Lamin A/C was detected by using the anti-lamin antibody (CST2032; Cell Signaling Technology) as a loading control. Note the loss of pCHK1 and total CHK1 (due to degradation) upon inhibition of ATR (lanes 4 and 6) or CHK1 (lane 5) but not inhibition of ATM (lanes 2 and 3), as reported recently (56). Conversely, pCHK2 was decreased by the ATM inhibitors (lanes 2, 3, and 6) but not ATR (lane 4) or CHK1 (lane 5), as expected. Download FIG S1, TIF file, 1.4 MB. Copyright © 2020 Luo et al. 2020 Luo et al. This content is distributed under the terms of the Creative Commons Attribution 4.0 International license.

10.1128/mBio.03423-19.2 FIG S2 ATR-CHK1 pathway could be activated by HBV replication in AML12HBV10 cells. HBV replication was induced in AML12HBV10 cells when the cells reached 70 to 80% confluence by removing tetracycline (Tet). (A) After 5 days of induction, immunofluorescence staining was performed on the induced (Tet–) and noninduced control (Tet+) cells using the pCHK1 specific antibody (S345, GTX 100065) or the HBc specific antibody (C1-5). The DAPI staining shows the cell nucleus. Staining was performed on 35-mm glass-bottom tissue culture dishes (MatTek), and the images were collected using a Leica SP8 confocal microscope. (B) After 1 day (D1), 5 days (D5), or 7 days (D7) of induction, the total cell lysate was analyzed by Western blotting with the pCHK1 specific antibody (S317, CST2344) (top) or the total CHK1 specific antibody (bottom). Download FIG S2, TIF file, 2.1 MB. Copyright © 2020 Luo et al. 2020 Luo et al. This content is distributed under the terms of the Creative Commons Attribution 4.0 International license.

10.1128/mBio.03423-19.3 FIG S3 ATR-CHK1 pathway could be activated by HBV infection in PXB cells. PXB cells were infected with HBV. Five days after infection, immunofluorescence staining was performed with the pCHK1 specific antibody (S317, CST12302). The DAPI staining shows the cell nucleus. Staining was performed on a 96-well plastic tissue culture plate (BD Bioscience), and the images were collected using a Nikon C2 confocal microscope. Download FIG S3, TIF file, 2.2 MB. Copyright © 2020 Luo et al. 2020 Luo et al. This content is distributed under the terms of the Creative Commons Attribution 4.0 International license.

The ATR-CHK1 pathway, once activated, can recruit multiple DNA repair effectors appropriate for the repair of different DNA damages (33, 42). Some of these factors could play important but redundant roles in the different steps of CCC DNA formation from RC DNA, e.g., completion of plus strand synthesis could be carried out conceivably by multiple DNA polymerases and ligation of the two strands by either ligase I or III (Fig. 9), consistent with reported findings so far (see the introduction). Future studies will be required to further elucidate how the ATR-CHK1 pathway is usurped by HBV to facilitate CCC DNA formation, a critical step in viral infection and persistence, and whether it can be exploited therapeutically to block CCC DNA formation and thus facilitate viral clearance.

## MATERIALS AND METHODS

### Cell cultures.

The AML12HBV10 cell line (39, 46), derived from an immortalized murine hepatocyte line AML12, and the HepAD38 cell line, derived from the human hepatoma cell line HepG2 (47), were maintained in the Dulbecco modified Eagle/F-12 medium (DMEM-F12) supplemented with 10% fetal bovine serum (FBS), 50 μg/ml of penicillin-streptomycin, 400 μg/ml G418, and 5 μg/ml of tetracycline (Tet). AML12HBV10 and HepAD38 cells were induced to express HBV pgRNA upon removal of Tet from the culture medium. The HepG2-NTCP cell line (32, 48) was derived from the human hepatoma cell line HepG2 and stably expresses the sodium taurocholate cotransporting polypeptide (NTCP), a functional receptor for HBV (49). HepG2-NTCP cells were maintained in DMEM-F12 supplemented with 10% FBS and 50 μg/ml of penicillin-streptomycin.

### HBV infection.

HBV infection of HepG2-NTCP was carried out as previously described (32). Briefly, the cells were plated in 35-mm dishes. When the cells reached 50% confluence, they were infected with the HBV inoculum harvested from HepAD38 cells (47) at a multiplicity of infection (MOI) of ca. 200 to 400 genome equivalents (GE)/cell in the presence of 2% dimethyl sulfoxide (DMSO) and 4% polyethylene glycol (PEG) 8000 in the presence or absence of the indicated compounds.

Infection of cryopreserved primary human hepatocytes (PHHs) was carried out as follows. One vial of PHHs (ca. 7.5 million cells) were thawed at 37°C within 2 min and then diluted in 20 ml of IVT thawing (HT) medium (BioIVT). The cells were then centrifuged at 80 × *g* for 5 min, and the supernatant was carefully discarded. The cells were resuspended with 5 ml of hepatocyte plating medium (BioIVT) and then seeded at 0.2 million/well onto collagen I-coated 24-well plates with the plating medium. On the following day, dead cells were removed by gentle shaking, and the plating medium was replaced with maintenance medium (DMEM-F12 containing 10% FBS, 1% DMSO, 100 U/ml penicillin, 100 μg/ml streptomycin, 5 ng/ml human epidermal growth factor, 50 nM dexamethasone, and 0.25 μg/ml insulin). Four hours later, infection was carried out by replacing the maintenance medium with infection medium (maintenance medium plus HBV inoculum from HepAD38 cells at an MOI of 500 GE/cell, 4% PEG 8000, and 1% DMSO; 150 μl per well). The next day, the cells were washed three times with warmed DMEM-F12 or phosphate-buffered saline (PBS) and 500 μl per well of matrix-containing maintenance medium was then added. The matrix-containing maintenance medium was prepared by thawing the Corning Matrigel Matrix at 4°C, followed by dilution to 0.25 mg/ml using cold maintenance medium. Thereafter, the culture medium was refreshed with maintenance medium every 2 days.

Freshly plated primary human hepatocytes isolated from chimeric mice containing human hepatocytes (PXB cell; PhoenixBio) were also used for HBV infection. PXB cells were plated on type I collagen-coated plates by PhoenixBio and cultured in modified dHCGM (DMEM with 10% FBS, 100 U/ml penicillin, 100 μg/ml streptomycin, 20 mM HEPES, 44 mM NaHCO_3_, 15 μg/ml l-proline, 0.25 μg/ml insulin, 50 nM dexamethasone, 5 ng/ml EGF, 0.1 mM Asc-2P, 2% DMSO) (41). Upon delivery, the cultured medium was replaced with fresh modified dHCGM. The cells were infected the next day (day 0) by replacing the culture medium with infection medium (dHCGM with 4% PEG 8000 and 5 μl of inoculum harvested from HepAD38 cells at an MOI of 400 GE/cell). The inoculum was incubated with the cells for 20 to 28 h. Subsequently, the culture medium was changed daily until day 3, when the cells were harvested for analysis of HBV CCC DNA.

### Inhibitors.

All chemical inhibitors were ordered from SelleckChem. These included the ATM inhibitors KU-55933 and KU-60019, the ATM/ATR dual inhibitors CGK733 and Torin2, the ATR inhibitors AZD6738 and VE-821, and the CHK1 inhibitors CHIR-124 and PF477736. All inhibitor stocks were made in DMSO with the following stocking concentrations: CGK733, 50 mM; KU-55933, 50 mM; AZD6738, 10 mM; Torin2, 10 mM; CHIR-124, 10 mM; PF477736, 20 mM; and VE-821, 50 mM.

### Determination of cytotoxicity.

Potential cytotoxicity of all compounds in each cell system was determined in pilot experiments in order to identify the highest compound concentration that did not induce significant toxicity, as judged by cell growth and morphological observation under light microscopy. The cytotoxicity of compounds on cryopreserved PHHs was further determined by using a CellTiter-Glo luminescent cell viability assay, which is based on measurement of cellular ATP content (Promega, Madison, WI). Briefly, PHHs (BioIVT) were seeded into collagen-coated 96-well plates at a density of 3 × 10^4^ cells per well and then incubated overnight under standard conditions to allow cell attachment. The culture medium was then removed, and the cells were exposed to the indicated concentrations of the compounds for 72 h. Cell lysate was then prepared according to the manufacturer’s instructions, and luminescence was read on a microplate reader (Envision 2104; Perkin-Elmer). Luminescence data were converted to growth fraction by comparison to the luminescence readout for the untreated control, and 50% inhibitory concentrations were determined from the graphical data (Fig. S4).

10.1128/mBio.03423-19.4 FIG S4 Cytotoxicity assay of DDR inhibitors in PHHs. The cryopreserved PHH cells were treated after plating with the indicated compounds for 72 h. Cell viability was then measured with a CellTiter-Glo luminescent cell viability assay (see Materials and Methods for details). Download FIG S4, TIF file, 0.3 MB. Copyright © 2020 Luo et al. 2020 Luo et al. This content is distributed under the terms of the Creative Commons Attribution 4.0 International license.

### Inhibitor treatments.

To inhibit the DDR pathways in HepG2-NTCP, PHH, or PXB cells during HBV infection, the indicated inhibitors were added along with the HBV inoculum. The cells were harvested 3 days later. To inhibit the DDR pathways in AML12HBV10 cells, the indicated inhibitors were added to the medium on day 4 after Tet removal. Three days later, the cells were harvested. To test the effect of DDR inhibition on intracellular CCC DNA amplification in the HepAD38 cells, we adopted the synchronized CCC DNA formation system, as reported recently (28). Briefly, Tet was removed from the culture medium to induce transcription of HBV pgRNA. On day 2 of Tet removal, the reversible inhibitor of the HBV reverse transcriptase, phosphonoformic acid (PFA) (50, 51), was added to the culture medium at 2 mM concentration to allow synthesis of HBV SS DNA but not RC DNA. PFA treatment was maintained for 4 days to accumulate nucleocapsids containing SS DNA. PFA was then removed to allow synchronous and rapid synthesis of RC DNA and the resulting conversion of RC to CCC DNA, and Tet was added back simultaneously to block further pgRNA transcription. At the time of PFA removal and Tet add back, the indicated DDR inhibitor was added. Cells were harvested 24 h after PFA removal for analysis of HBV DNA.

### siRNA knockdown.

The CHK1 ON_TARGET siRNA pools and negative-control siRNA were purchased from Dharmacon. HepG2-NTCP cells were plated on 35-mm dishes on day 1 at a density so they reached ca. 20% confluence on day 2. Cells were transfected with 25 nM siRNA and DharmaconFECT 4 on day 2 and again on day 3. The cells were infected on day 4 with HBV and harvested on days 6 and 7 (i.e., days 2 and 3 postinfection).

### Antibodies.

The rabbit polyclonal antibody (pAb; CST 2344) and rabbit monoclonal antibody (MAb; CST 12302), both specific for the phosphorylated CHK1 (pCHK1) phosphorylated on S317, were ordered from Cell Signaling Technology (CST). The rabbit pAb specific for pCHK1 phosphorylated on Ser345 (GTX100065) was purchased from GenTex. The mouse MAb specific for total CHK1 (AM7401a) was ordered from Abgent. The mouse MAb specific for HBc (C1-5) was obtained from Santa Cruz. The rabbit pAb specific for β-actin (CST 4967) was obtained from CST.

### Immunofluorescence assay.

Cells cultured on glass-bottom 12-well plates or 35-mm glass-bottom dishes (MatTek) were washed three times with PBS. The cells were then fixed with freshly prepared 4% paraformaldehyde for 15 min at room temperature. The fixed cells were washed three times with 30 mM glycine in PBS (pH 7.4) and then permeabilized with 0.1% Triton X-100 for 5 min at room temperature. The cells were washed again with PBS three times and then incubated with the blocking buffer (3% bovine serum albumin in PBS with 0.1% Triton X-100) for 1 h at room temperature. The cells were then incubated with the indicated primary antibody diluted in blocking buffer at 1:100 at 4°C overnight. The next day, the cells were washed three times with PBS and incubated with the appropriate fluorescence-labeled secondary antibody for 1 h at room temperature. The cells were then washed three times with PBS. Glass Bottom Fluid (MatTek) was then applied to take off the glass coverslips from the culture plates or dishes. The coverslips were subsequently mounted on slides in mounting medium containing DAPI (4′,6′-diamidino-2-phenylindole; Vector Laboratories). Images were collected using a Leica SP8 confocal microscope. The primary antibodies used were anti-HBc (C1-5) at a 1:100 dilution, anti-pCHK1 (S345 GTX 100065) at 1:100 for AML12HBV10 cells, and anti-pCHK1 (CST12302) at 1:100 for HepG2-NTCP and PXB cells. The secondary antibodies used were goat anti-rabbit Alexa 546 (1:2,000) and goat anti-mouse Alexa 488 (1:2,000).

### Isolation of HBV DNA.

HBV core DNA (nucleocapsid [NC]-associated DNA) and PF DNAs were isolated as previously described (29, 32, 52), with minor modifications. For isolation of core DNA, cells were lysed in NP-40 lysis buffer (50 mM Tris-HCl [pH 8.0], 1 mM EDTA, 1% NP-40, and 1× protease inhibitor [Roche]). After removal of the nuclear pellet by centrifugation, the supernatant (cytoplasmic lysate) was incubated with micrococcal nuclease (MNase; Roche) at 150 U/ml and 5 mM CaCl_2_ at 37°C for 90 min to degrade the nucleic acids outside NCs. The MNase was then inactivated by addition of 10 mM EDTA. Proteinase K (final concentration, 0.6 mg/ml) and sodium dodecyl sulfate (SDS; 0.5%) were then added to digest and disrupt viral DNA-protein complexes, which were then resolved by agarose gel electrophoresis. A modified Hirt extraction was used for PF DNA isolation (53). Briefly, cells in a 60-mm dish were lysed in 1 ml SDS lysis buffer (50 mM Tris-HCl [pH 8.0], 10 mM EDTA, 150 mM NaCl, and 1% SDS). After incubation for 5 min at room temperature, the cell lysate was transferred to a 1.5-ml microcentrifuge tube, mixed with 0.25 ml of 2.5 M KCl, and incubated at 4°C overnight with gentle rotation. After spinning at 14,000 × *g* for 20 min, the supernatant was extracted three times with phenol and once with chloroform. The DNA was precipitated with ethanol and washed with 70% ethanol three times, vacuum dried, resuspended in 200 μl of TE (10 mM Tris-HCl–1 mM EDTA [pH 8.0]).

### Exonuclease treatment.

For exonuclease I (Exo I) and Exo III digestion, 20 μl of PF DNA was digested with 1 μl of Exo I (20 U) and 0.25 μl of Exo III (25 U) in 1× Cutsmart Buffer (NEB) at 37°C for 2 to 3 h (32). The DNA was then loaded onto an agarose gel for Southern blot analysis or extracted with phenol and chloroform and purified before PCR analysis.

### Southern blot analysis of HBV DNA.

Agarose gel electrophoresis and Southern blot analysis were performed as previously described (32, 51, 54). HBV DNA was detected by using ^32^P-labeled HBV DNA probe or strand-specific RNA probe (riboprobe) as indicated.

### Quantification of total HBV PF DNA and CCC DNA from PHHs by quantitative PCR.

HBV PF DNA from infected PHHs was extracted by Hirt extraction and digested with Exo I and Exo III as described above. Two microliters of digested and purified PF DNA (10% of purified PF DNA from one well of a 24-well plate) was then mixed with 500 nM concentrations of primers, a 100 nM concentration of probe, and 10 μl of LightCycler 480 probe master mix reagent (Roche). Sterile dH_2_O was added to bring the total volume to 20 μl per reaction. Quantitative PCR (qPCR) was performed on a LightCycler 480 II system on 384-well plates. The cycling condition was 95°C for 10 min and then 95°C for 10 s, 58°C for 5 s, 63°C for 10 s, and 72°C for 40 s for a total of 45 cycles. The pBR322-HBV1.3 plasmid containing a 1.3-mer HBV genome was serially diluted and used as qPCR standards (55). The primer and probe sequences for total HBV DNA amplification were as follows: forward primer, 5′-GCTGGATGTGTCTGCGGC-3′ (positions 372 to 389); reverse primer, 5′-GAGGACAAACGGGCAACATAC-3′ (459 to 479); and probe, 5′-TAMRA+CATCCTGCTGCTATGCCTCATCTTCTTG+BHQ-2-3′ (409 to 436). The primer sequences for CCC DNA amplification were as follows: forward (1529 to 1549), ACCTCTCTTTACGCGGACTCC; reverse (2097 to 2118), CCCACCCAGGTAGCTAGAGTCA; and probe, 5′-TAMRA+ATTGGTCTGCGCACCAGCACCA+BHQ-2-3′ (1793 to 1814).

### Quantification and statistical analysis.

All experiments were repeated at least three times. DNA signals from Southern blot analysis were detected by phosphorimaging using a Typhoon 9500 scanner (GE Healthcare Life Sciences) and quantified using Quantity One software (Bio-Rad). Protein signals from Western blot analysis were detected and quantified using the Bio-Rad Chemi-Doc (ImageLab) system. Statistical analysis was performed using the two-tailed, unpaired Student *t* test with Excel.
