# Supplementary figures and images for: Involvement of Host ATR-CHK1 Pathway in Hepatitis B Virus Covalently Closed Circular DNA Formation
Source: mBio. 2020 Feb 18;11(1):e03423-19. doi: 10.1128/mBio.03423-19 (PMC7029148; doi:10.1128/mBio.03423-19)

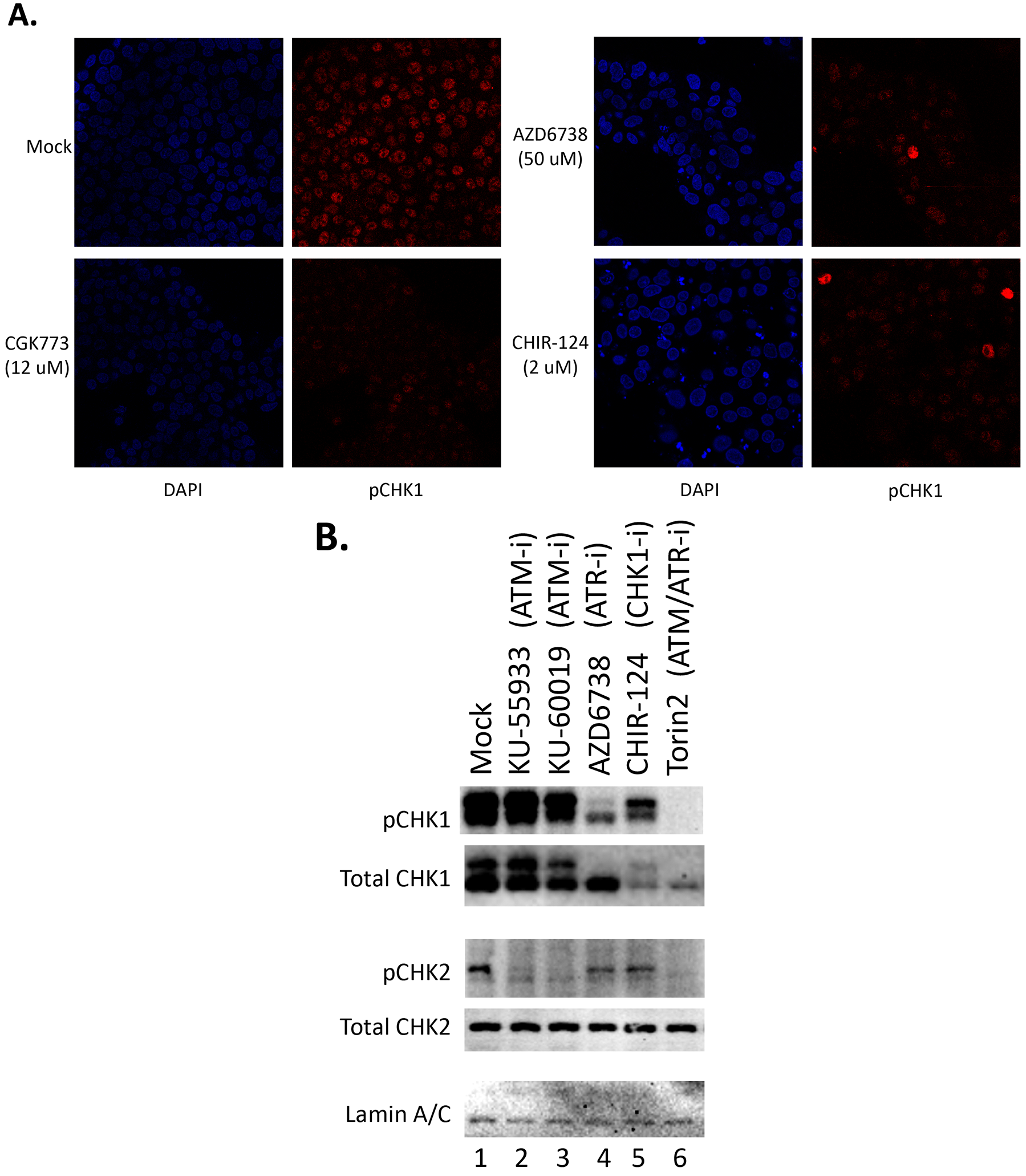

Supplement: FIG S1 [file mBio.03423-19-sf001.tif]

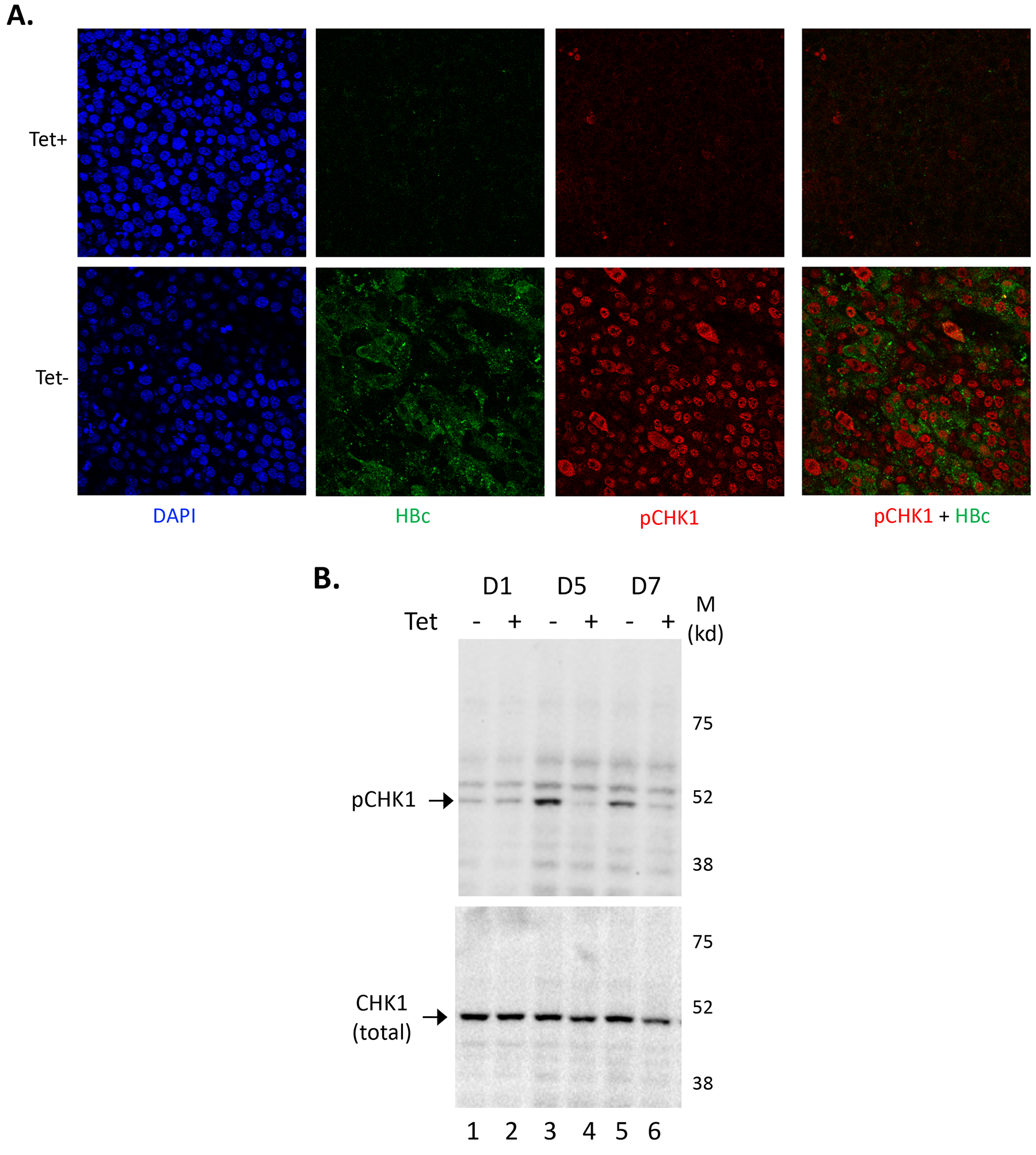

Supplement: FIG S2 [file mBio.03423-19-sf002.tif]

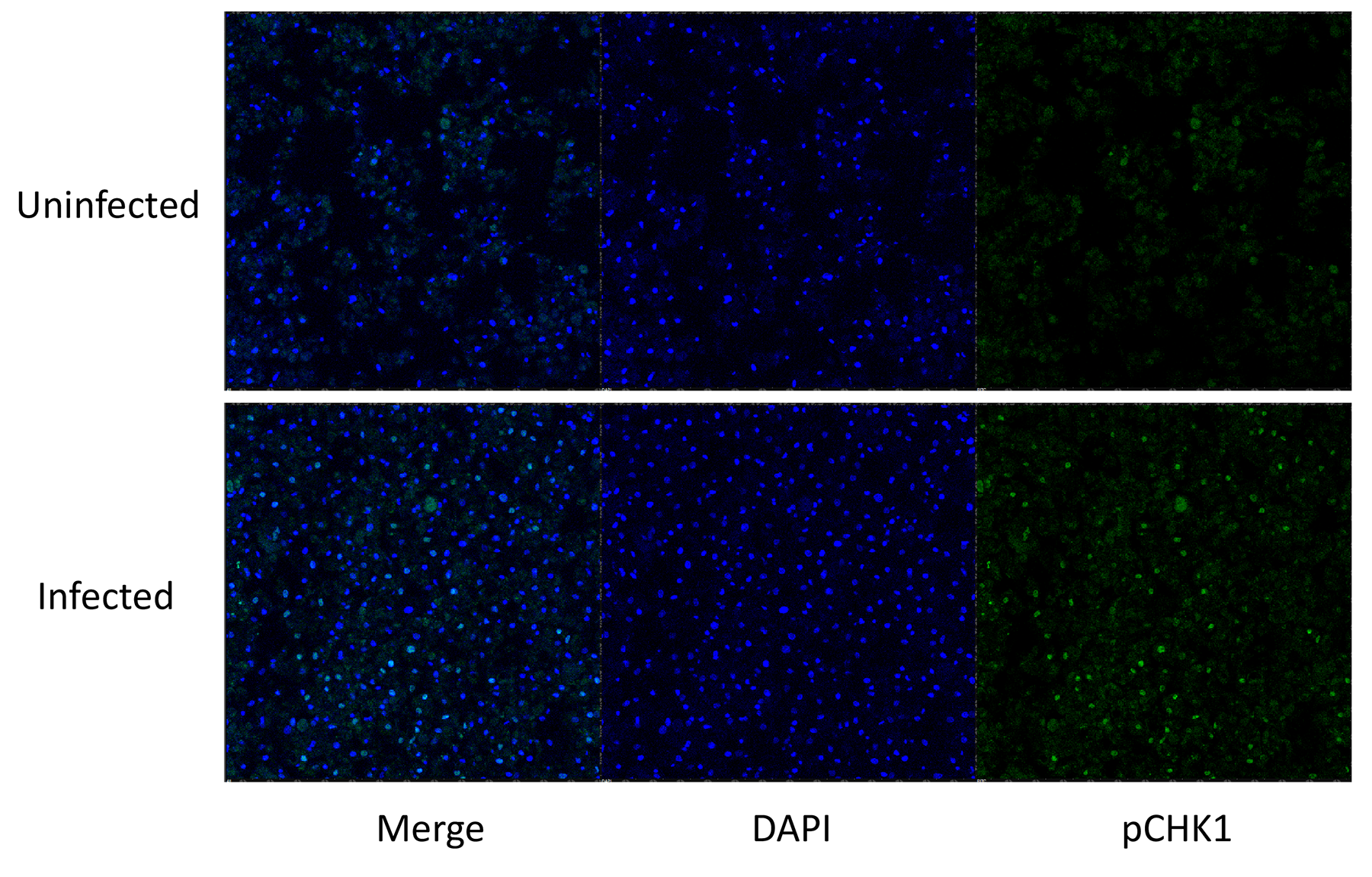

Supplement: FIG S3 [file mBio.03423-19-sf003.tif]

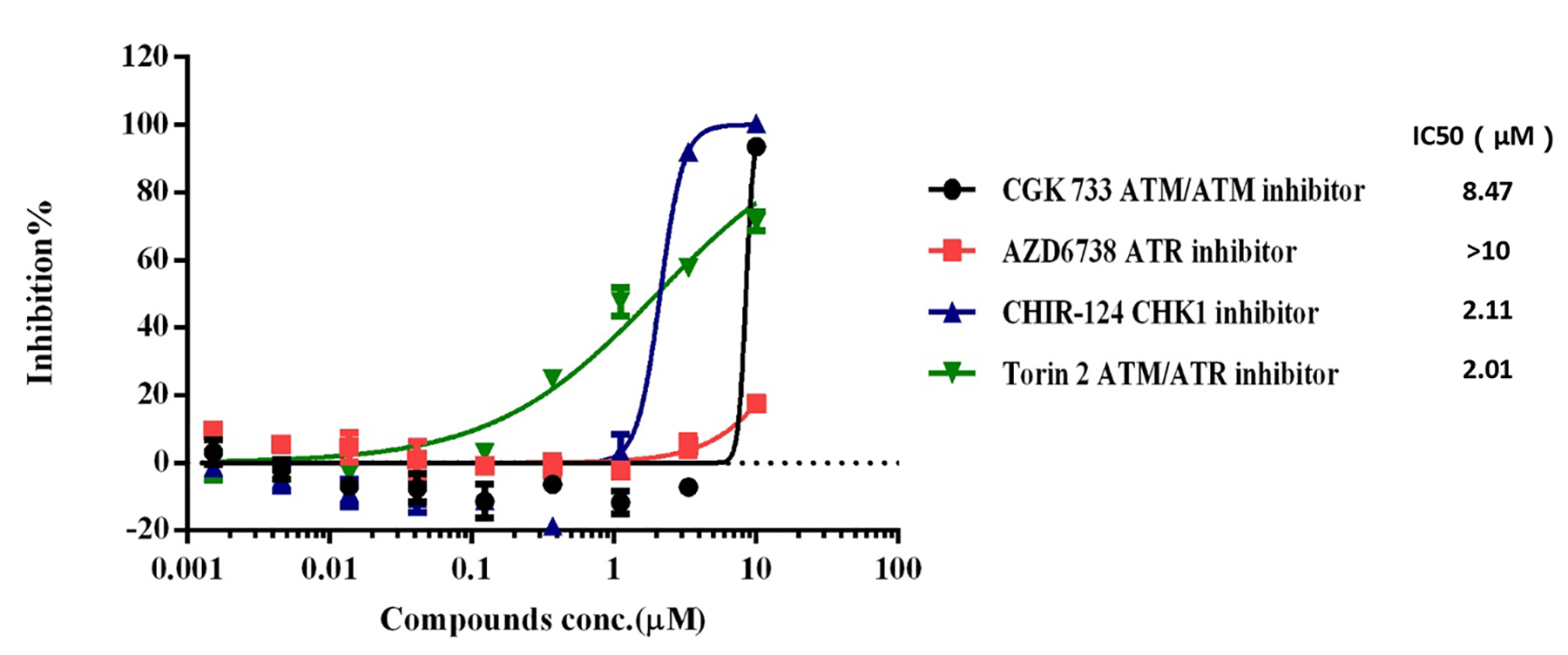

Supplement: FIG S4 [file mBio.03423-19-sf004.tif]
